# National Health Insurance interprofessional practice implementation in hand rehabilitation service delivery in South Africa

**DOI:** 10.4102/sajp.v80i1.1969

**Published:** 2024-04-23

**Authors:** Monique M. Keller

**Affiliations:** 1 Department of Physiotherapy, Faculty of Health Sciences, University of the Witwatersrand, Parktown, South Africa

**Keywords:** physiotherapy, National Health Insurance, hand therapy, interprofessional practice, interprofessional education, International Classification of Functioning, Disability and Health, ICF core sets

## Abstract

South Africa’s current healthcare system is unequal and inefficient, with 71% of the population accessing underfunded public healthcare facilities. The imminent National Health Insurance (NHI) aims to balance the inequality between private and public health services by providing universal healthcare. The high levels of road accidents, violence and injuries in South Africa, being one of the quadruple burdens of diseases frequently causing hand injuries with resultant devastating economic and functional implications without rehabilitation services, are concerning. The plea is that for specialised injuries, such as hand injuries, the NHI implementation requires innovative interprofessional health education and practice when managing individuals who sustained hand injuries optimally to return to preinjury functioning. When considering the devastating burden of mismanaged hand injuries on all levels of the International Classification of Functioning, Disability and Health (ICF) framework, all South Africans deserve rehabilitation by physiotherapists and occupational therapists based on each occupation’s unique strengths. Anecdotally, a lack of interprofessional undergraduate and postgraduate hand rehabilitation education and hand-injured patient management exists in South Africa, highlighting the need for urgent change towards future practice and NHI. This commentary aims to apply the ICF core sets to inform interprofessional hand rehabilitation delivery by physiotherapists and occupational therapists.

**Clinical implication:**

Interprofessional rehabilitation framed in the ICF Core Set, accompanied by inclusive interprofessional education opportunities according to the Health Professions Council of South Africa (HPCSAs) scope of practice, will benefit South Africans who sustained hand injuries to fully recover and maximise their functional performance considering the type of injuries sustained.

## Introduction

Hand injuries typically affect the young, working and economically productive population (De Putter et al. 2012) and account for up to 30% of all emergency department consultations globally, with 6.6% (Hill et al. 1998) to 28.6% (Angermann & Lohmann 1993) of all injuries in the hand and wrist. In South Africa, Naidoo, Govender and Naidoo (2021) confirm that traumatic injuries account for 17.8% of all emergency consultations in the KwaZulu-Natal province, with injuries frequently occurring on the hand. It is concerning because hand injuries impact individuals’ abilities to perform work, self-care and leisure activities (World Health Organization 2007). Therefore, the International Classification of Functioning’s activity and participation restriction domains are impacted negatively with economic consequences (Robinson et al. 2016) to the individual and the larger society.

South African health professionals deliver services in different contexts and a complex healthcare environment, necessitating a collaborative approach. Khan and Sivakumar (2016) agree that a multidisciplinary team, where surgeons, occupational therapists and physiotherapists work in a coordinated approach to provide patient care for the often lengthy recovery period following hand injuries, is needed. The multidisciplinary team members involved in managing individuals with hand injuries include, but are not limited to, orthopaedic surgeons, plastic surgeons, medical officers, clinical associates, rheumatologists, emergency medicine physicians, radiologists, radiographers, pharmacists, prosthetists and orthotists, nurses, physiotherapists and occupational therapists. For the aim of this article, physiotherapists and occupational therapists working as an interprofessional team, predominantly responsible for delivering hand rehabilitation, will be focused on.

The problem is that the physiotherapists’ role in hand rehabilitation, often also called hand therapy, in the past two decades has decreased in importance because of the lack of postgraduate education opportunities and the incorrect and unfounded anecdotal narrative that ‘physiotherapists don’t see hand patients’, which is not the instance internationally. With the already underserviced, unequal health services delivery in South Africa’s private, but more pertinently, public and rural areas, an immediate need exists to correct past narratives and perceptions (Keller 2023). This commentary further aims to position physiotherapists in South Africa as primary health practitioners in the intraprofessional and multidisciplinary team and towards preparation for the National Health Insurance (NHI) in the interprofessional team delivering hand rehabilitation. Achieving this aim may improve hand rehabilitation service delivery to optimise hand function and quality of life for individuals who sustained hand injuries.

To clarify definitions of teamwork approaches, interprofessional teams comprise healthcare professionals from different disciplines working together to provide comprehensive patient care. In managing patients who sustained hand injuries, the teams typically include occupational therapists, physiotherapists, hand surgeons, plastic surgeons and other healthcare professionals who work together to provide coordinated care to patients with hand injuries or conditions. Interprofessional teams in hand rehabilitation work to improve patient outcomes by providing comprehensive, coordinated care that addresses the whole person’s needs. Intraprofessional teams, on the other hand, refer to teams composed of healthcare professionals from the same discipline who work together to provide specialised care. Multidisciplinary teams are composed of healthcare professionals from different disciplines who work together, not necessarily in a coordinated manner, to provide specialised care to patients with hand injuries or conditions.

The commentary firstly presents physiotherapy as a profession delivering hand rehabilitation according to the Health Professions Council of South Africa (HPCSA) requirements. After that, the NHI is introduced. The ICF framework follows with the core sets specifically related to hand conditions included (Cieza et al. 2004). In table format, the author indicates, referring to a past interprofessional practice in a South African Hand Unit, what physiotherapy and occupational therapists’ roles are in the interprofessional team delivering optimal hand rehabilitation.

## Physiotherapy profession delivering hand rehabilitation

According to the HPCSA, a physiotherapist is responsible for evaluating, addressing and overseeing a diverse range of injuries, including but not limited to orthopaedic conditions such as hand injuries. The scope of practice for South African physiotherapists includes the entire field of orthopaedics, including dislocations, fractures, soft tissue and ligament injuries, bone infections, amputations, joint diseases and deformities, with special mention to the inclusion of assessment and treatment of specialised areas of hand surgery and muscle and tendon transplants with various modalities and tools including splints and prostheses (*Health Professions Act 56 of 1974*; HPCSA Scope of Physiotherapy 1976). The HPCSA Professional Board for Physiotherapy, Podiatry and Biokinetics’ undergraduate minimum standards of training of physiotherapy students, which was updated on 20 March 2023, confirms that undergraduate trainings at tertiary institutions are to include knowledge and skill training in splinting and braces as assistive and supportive devices to name one aspect, to equip students to manage patients effectively.

According to the HPCSAs’ rationale for the physiotherapy profession, physiotherapy as a healthcare profession aims to offer services to individuals and communities to cultivate, sustain and reinstate optimal movement and functionality throughout the person’s life. The HPCSA further underpins physiotherapy by referring to the ICF framework in the following statement:

Physiotherapy is provided for individuals who have, or may develop impairments, activity limitations, and participation restrictions, related to conditions of the neuromusculoskeletal, neurological, cardiovascular, pulmonary, and/or integumentary systems as they relate to human movement, or due to personal and environmental factors. (HPCSA Minimum standards of training 2023)

An additional qualification for physiotherapists, namely the postgraduate diploma in hand therapy, is included (*Health professions Act 56 1974*). The primary focus of physiotherapy thus lies in recognising and enhancing the quality of life and the potential for movement within the entire spectrum of healthcare, encompassing prevention, promotion, treatment, rehabilitation, intervention and referral (HPCSA Minimum Standards of Training 2023). Physiotherapists have an instrumental role in the proposed and implementation of the NHI.

## National Health Insurance

On 12 June 2023, the South African Government National Assembly passed the NHI Bill in the plenary sitting, aiming to provide all South Africans with universal access to healthcare services. The fund envisages purchasing healthcare services for all the users for effective, efficient and equitable resource utilisation and addresses the barriers South Africans encounter when accessing healthcare (Parliament of the Republic of South Africa press release 2023). The NHI will offer individuals across all income levels to seek medical care at public and private hospitals and services, and the NHI pays the private and public medical professionals equally. Physiotherapy and occupational therapy professions both need to be positioned to deliver interprofessional hand rehabilitation for individuals who sustained hand injuries towards the NHI. To achieve excellent patient outcomes, according to my experience, the best approach is to utilise the strengths of both professions in the hand rehabilitation for these injured individuals. Equal opportunity to education and interprofessional hand rehabilitation education is imperative for the future of hand rehabilitation delivery in South Africa towards NHI implementation (Keller 2023). The ICF framework best underpins hand rehabilitation interprofessional practice.

## International Classification of Functioning, Disability and Health

**FIGURE 1:**
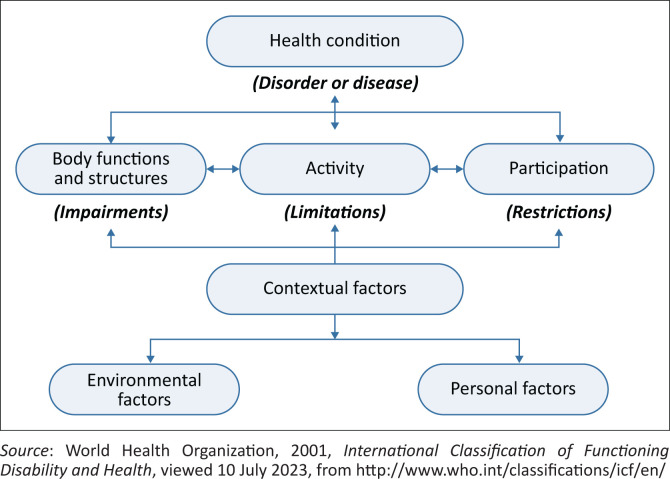
Interaction between the components of the International Classification of Functioning.

The ICF system categorises health and health-related domains to describe an individual’s functioning and disability within a specific context. It also includes environmental factors (World Health Organization 2001). All World Health Organization member states officially endorsed the ICF as the gold standard for describing and measuring disability and health at both individual and population levels on 22 May 2002 (World Health Organization 2001). In South Africa, hand injuries resulting in decreased functioning of a dominant or non-dominant hand can leave individuals unable to earn a living, increasing the burden of poverty on society. The primary outcome of hand rehabilitation should be a fully functional hand to ensure the earliest return to work. Successful hand rehabilitation should address the three ICF areas, which include body function and structure, activity limitations and participation restrictions, as well as contextual factors, such as environmental and personal factors, which may influence an individual’s functioning (Functioning and Disability Reference Group 2010; World Health Organization 2001). The ICF framework, in Figure 1, is fundamental and the critical considerations in holistic management of individuals who sustained hand injuries.

Stucki (2005) notes that, except for personal factors unique to each individual, the ICF model components are classified in a standardised manner to provide a universal description and understanding of health-related conditions and general health. Hand conditions such as fractures, tendon injuries, crush injuries and vascular or neurological injuries can negatively impact the three ICF levels and contextual factors, such as an individual’s personal factors, community and environment. Personal factors, including race, age, coping styles, smoking status, gender and preexisting medical conditions such as diabetes mellitus type I and type II, osteoporosis, adherence to health practitioners’ advice and prescriptions and renal failure (Wollstein et al. 2020), play an essential role in managing injuries like fractures and should be considered by clinicians. Moreover, biopsychosocial impacts can occur when considering physical and psychological challenges to functioning and performing daily tasks without a dominant and nondominant hand. Psychological challenges that precede injury can also linger unseen, resulting in disability (Coovadia et al. 2009; Roberts et al. 2009). Activity limitations, classified in the ICF, are caused by poor hand function resulting from the inability to grasp and pinch, usually after a hand injury. Interprofessional practice in hand rehabilitation may allow the individual to rehabilitate on all the ICF framework domains fully.

A personal experience of working in an interprofessional team delivering hand rehabilitation in South Africa in the hand unit at a public hospital in Soweto, Gauteng will now be presented. Occupational therapists and physiotherapists deliver different aspects of hand rehabilitation per the unique individualised needs of each patient visiting the hand unit according to each profession’s strengths. Communication and respect for each team member ensured the patients and their needs according to the ICF framework, informed the hand rehabilitation delivery. In-service training between therapists of the same profession and interprofessional education in the form of morning meetings brought the orthopaedic surgeons, plastic surgeons, registrars, interns, occupational therapists and physiotherapists together to share experiences. Informal, unstructured interprofessional learning occurred during presentations of patients’ cases, management options and considerations, clinical reasoning and decision making. The impairments, limitations and restrictions of ICF Core Set components for hand conditions (ICF Research Branch 2017), which were managed interprofessionally, but indicating which occupation took primary responsibility for each ICF Core Set can be found in Table 1. Table 1 indicates where physiotherapy (no italic), occupational therapy (italic) and where no distinction could be made, but both professions could consider the ICF Core Set (bold) managed injured individuals according to the ICF framework.

**TABLE 1:** International Classification of Functioning core set for hand conditions per domain.

Body functions and structures (impairments)		Activity (limitations)		Participation (restrictions)	
ICF code	Title	ICF code	Title	ICF code	Title
B260	Proprioceptive function	*D170*	*Writing*	*D840–D859*	*Work and employment*
*B265*	*Touch function*	*D230*	*Carrying out daily routine*	*D920*	*Recreation and leisure*
*B2700*	*Sensitivity to temperature*	**D360**	**Using communication devices and techniques**	**E310**	**Immediate family**
*B2701*	*Sensitivity to vibration*	**D410**	**Changing basic body position**	**E315**	**Extended family**
*B2702*	*Sensitivity to pressure*	*D420*	*Transferring oneself*	**E320**	**Friends**
B280	Sensation of pain	**D430**	**Lifting and carrying objects**	-	-
B7100	Mobility of a single joint	**D4400**	**Picking up**	-	-
B7101	Mobility of several joints	*D4401*	*Grasping*	-	-
B715	Stability of joint functions	*D4402*	*Manipulating*	-	-
B720	Mobility of bone functions	*D4403*	*Releasing*	-	-
B7300	Power of isolated muscles and muscle groups	*D4408*	*Fine hand use, other specified*	-	-
B7301	Power of muscles of one limb	*D4450*	*Pulling*	-	-
Bb735	Muscle tone functions	*D4451*	*Pushing*	-	-
B740	Muscle endurance functions	*D4452*	*Reaching*	-	-
**B760**	**Control of voluntary movement functions**	*D4453*	*Turning or twisting the hands or arms*	-	-
**B765**	**Involuntary movement functions**	**D4454**	**Throwing**	-	-
B810	Protective functions of the skin	**D4455**	**Catching**	-	-
*B820*	*Repair functions of the skin*	*D4458*	*Hand and arm use, other specified*	-	-
*B840*	*Sensation related to the skin*	*D475*	*Driving*	-	-
S720	Structure of shoulder region	*D510*	*Washing oneself*	-	-
S7300	Structure of the upper arm	*D520*	*Caring for body parts*	-	-
S7301	Structure of the forearm	*D530*	*Toileting*	-	-
**S7302**	**Structure of the hand**	*D540*	*Dressing*	-	-
S770	Additional musculoskeletal structures related to movement	*D550*	*Eating*	-	-
D410	Changing basic body position (posture correction)	*D560*	*Drinking*	-	-
		*D630*	*Preparing meals*	-	-
		*D640*	*Doing housework*	-	-
		*D660*	*Assisting others*	-	-

*Source:* ICF Research Branch, 2017, *Comprehensive ICF Core Set for Hand Conditions*, viewed 11 July 2023, from https://www.icf-research-branch.org/images/ICF%20Core%20Sets%20Download/Comprehensive_ICF_Core_Set_for_Hand_Conditions.pdf

Note: Bold font indicates where both professions play a role in a specific core set item.

ICF, International Classification of Functioning.

## Recommendations

It is recommended that health professionals in South Africa’s private, public and rural facilities managing hand-injured individuals should determine the available interprofessional team members and have contextually relevant discussions. The discussion includes but is not limited to the ICF Core Set for hand condition rehabilitation allocation to guide patient management and rehabilitation. This should be supported by interprofessional education at undergraduate and postgraduate levels in South African tertiary institutions, both formally and informally. All South Africa’s private, public and rural facilities’ stakeholders should be involved in discussing, planning and implementing interprofessional education to support the current practice and future needs of the NHI system. The hand rehabilitation training that physiotherapists receive at an undergraduate level forms the foundation for providing rehabilitation; however, continued education is needed formally or informally at a postgraduate level. The responsibility, therefore, lies with each interprofessional health practitioner to stay abreast of the latest hand injury management evidence informing clinical practice. The HPCSA allows health professionals to utilise management tools when they are trained, skilled and experienced in these management tools. Additionally, future hand rehabilitation research should focus on determining the effectiveness of an implemented interprofessional practice approach and the effect of interprofessional education in undergraduate and postgraduate hand rehabilitation curricula.

## Conclusion

This strategic, cohesive, interprofessional approach will ensure a comprehensive and collaborative approach to managing hand injuries and supporting the development of healthcare professionals in South Africa towards improving current patient management and NHI implementation. This is where interprofessional practice and education in South Africa, in a team, ultimately benefits the patients.
